# Predictors of Disease-Free and Overall Survival in Retroperitoneal Sarcomas: A Modern 16-Year Multi-Institutional Study from the United States Sarcoma Collaboration (USSC)

**DOI:** 10.1155/2019/5395131

**Published:** 2019-06-02

**Authors:** Patrick B. Schwartz, Kara Vande Walle, Emily R. Winslow, Cecilia G. Ethun, Thuy B. Tran, George Poultsides, Jennifer Tseng, Kevin Roggin, Valerie Grignol, John Harrison Howard, Bradley A. Krasnick, Ryan C. Fields, Harveshp Mogal, Callisia N. Clarke, Rebecca Senehi, Konstantinos Votanopoulos, Kenneth Cardona, Daniel E. Abbott

**Affiliations:** ^1^University of Wisconsin, Madison, WI, USA; ^2^Emory University, Atlanta, GA, USA; ^3^Stanford University, Stanford, CA, USA; ^4^University of Chicago, Chicago, IL, USA; ^5^The Ohio State University, Columbus, OH, USA; ^6^Alvin J. Siteman Cancer Center, Washington University School of Medicine, St. Louis, MO, USA; ^7^Medical College of Wisconsin, Milwaukee, WI, USA; ^8^Wake Forest University, Winston-Salem, NC, USA

## Abstract

**Background:**

Retroperitoneal sarcomas (RPS) comprise approximately 15% of all soft-tissue sarcomas and frequently associated with significant morbidity and as little as 30% 5-year survival. Here, we provide a large, contemporary, and multi-institutional experience to determine which tumor, patient, and treatment characteristics are associated with long-term outcomes in RPS.

**Methods:**

571 patients with primary RPS were identified from the United States Sarcoma Collaboration (USSC). RPS patients who underwent resection from January 2000 to April 2016 were included with patient, tumor, and treatment-specific variables investigated as independent predictors of survival. Survival analyses for disease-free and overall survival were conducted using Kaplan–Meier and Cox proportional hazards model methods.

**Results:**

The study cohort was 55% female, with a median age of 58.9 years (IQR: 48.6–70.0). The most common tumor histiotypes were liposarcoma (34%) and leiomyosarcoma (28%). Median follow-up was 30.6 months (IQR: 11.2–60.4). Median disease-free survival was 35.3 months (95% CI: 27.6–43.0), with multivariate predictors of poorer disease-free survival including higher grade tumors, nodal-positive disease, and multivisceral resection. Median overall survival was 81.6 months (95% CI: 66.3–96.8). Multivariate predictors of shorter overall survival included higher grade tumors, nodal-positive and multifocal disease, systemic chemotherapy, and grossly positive margins (*R*2) following resection.

**Conclusions:**

The strongest predictors of disease-free and overall survival are tumor-specific characteristics, while surgical factors are less impactful. Nonsurgical therapies are not associated with improved outcomes despite persistent interest and utilization. Complete macroscopic resection (*R*0/*R*1) remains a persistent potentially modifiable risk factor associated with improved overall survival in patients with retroperitoneal sarcomas.

## 1. Introduction

Sarcomas are tumors of mesenchymal origin and represent only approximately 1% of all cancers in the adults. These tumors are most commonly found in the extremity; however, they are retroperitoneal in 15% of cases [1]. Despite their rarity, sarcomas represent a vast array of tumor histiotypes, currently with 60 identified subtypes, with behavioral differences making study and generalizations about their care difficult [2].

Retroperitoneal sarcomas (RPS) in particular, due to their location, tend to be asymptomatic and appear at late stages and with historically poor outcomes; the average tumor size at diagnosis can be up to 15–20 cm [3]. The rarity of these tumors and number of histiotypes have led to recent attempts to consolidate investigative efforts between institutions, such as with the Transatlantic RPS Working Group (TAWG), Surveillance, Epidemiology, and End Results (SEER), and the National Cancer Database (NCDB) [4–6].

Previous studies report that the strongest and most consistent predictor of either disease-free or overall survival is tumor grade [7, 8]. Larger tumor size, although variably defined, has also been shown to be predictive of worse survival outcomes [4, 5]. Furthermore, margin status appears to contribute to long-term outcomes as gross residual disease following resection has been associated with survival similar to unresectable disease (though some suggest long-term survival can be achieved with a *R*2 resection in select circumstances with favorable histological subtypes) [9–11]. And finally, the utility of chemotherapy or radiotherapy in RPS continues to be poorly understood (TAWG). The importance of nonsurgical therapies remains an active focus of investigation, with a recent study suggesting possible overall survival benefit with administration of radiation therapy and recent European trials showing benefits to epirubicin- and ifosfamide-based chemotherapy regimens for soft-tissue extremity and truncal sarcomas [12, 13]. Finally, the use of histologically target-based chemotherapies has increased and continues to be an ongoing and active area of research [14].

Using a contemporary database with granular histopathologic data, the purpose of this investigation was to identify factors associated with recurrence-free survival and overall survival in patients with retroperitoneal sarcomas. Our primary aim was to discern whether any modifiable pre-, intra-, or postoperative patient-, provider-, or tumor-specific characteristics could be identified. Additionally, we sought to determine if trends in increasing use of chemotherapy and radiotherapy had any effect on outcomes. The granularity of the histopathologic data from the USSC database in conjunction with its size makes it poised to add to the growing body of the literature attempting to identify factors which predict worse survival.

## 2. Methods

### 2.1. Patient Population

The study cohort was derived from the United States Sarcoma Collaboration (USSC) database, created from the following tertiary centres: University of Wisconsin, Emory University, Stanford University, Medical College of Wisconsin, Wake Forest University, the Ohio State University, University of Chicago Medicine, and Washington University. A total of 571 patients with primary retroperitoneal sarcomas (RPS) treated between January 2000 and April 2016 was included. Only primary RPS were considered, with recurrent tumors excluded. All patients underwent resection, and follow-up was recorded in months. Histologic diagnosis was confirmed, and grade was assigned as either low grade (Federation Nationale des Centres de Lutte Contre le Cancer (FNCLCC) = *G*1), high grade (FNCLCC = *G*2 or *G*3), or unable to be assessed (FNCLCC = *G*X) according to the FNCLCC or TNM two-tier grading schema [15, 16].

In this retrospective cohort analysis, independent variables included age, sex, smoking status, prior radiation exposure, genetic syndromes, tumor size, nodal disease, multifocal disease, number of organs included in an en bloc resection, receipt of neo- or adjuvant chemotherapy/radiation, and postoperative margin status. Primary outcomes of interest were disease-free survival and overall survival. Secondary outcomes were trends of survival over time. Recurrence was defined as either pathologic or radiographic evidence of recurrence following resection, in months. Overall survival was defined as the length of time between surgery and death from any cause with time censored at last follow-up.

### 2.2. Statistical Analysis

All continuous variables were reported as median values with an associated interquartile range. All categorical variables were reported as a percentage of the total. Categorical comparisons were made using chi-squared test or Fisher's exact test (FET) as appropriate. Univariate Kaplan–Meier analysis using logrank testing and subsequent multivariate analysis utilizing Cox proportional hazards ratios were derived for the outcomes of interest. All *p* values <0.05 were considered significant in univariate analysis and thus included in multivariate analyses. All *p* values <0.05 in multivariate analyses were considered significant. Univariate subanalysis was performed to determine if differences existed between tumor histiotype and recurrence-free survival and overall survival and locoregional versus distant recurrence. Additional multivariate logistical regression was performed, where appropriate, on selected subanalysis thought to not replicate prior analyses. Multivariate logistical regression analysis was performed in a similar fashion to the Cox proportional hazards analysis. All data were stored using Excel (Microsoft Corporation, Redmond, WA), and all statistical analysis was performed using SPSS 24 (IBM Corporation, Armonk, NY). Appropriate IRB approval was obtained from each institution.

## 3. Results

### 3.1. Patient Demographics

The study population was 45% male and predominantly white (72%). Median BMI was 27 (IQR: 27.0–31.7). Median age was 58.9 (interquartile range (IQR): 48.6–70.0), with other demographics outlined in Table 1. Median follow-up was 30.6 months (IQR: 11.2–60.4). Three percent of patients had a known genetic syndrome, including NF1, Li–Fraumeni, and FAP.

**Figure 1:**
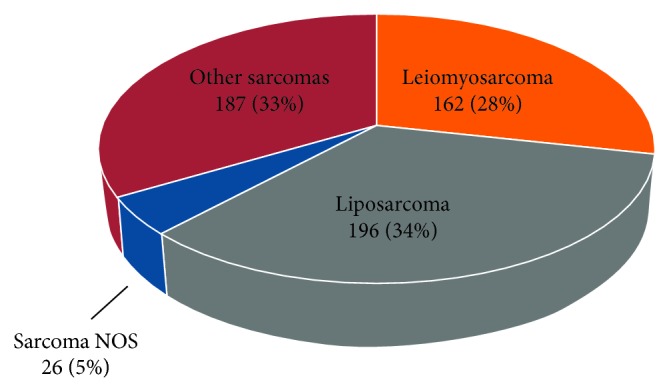
Pie chart demonstrating proportion of different histological subtypes.

The most common tumor histiotype was liposarcoma (34%), followed by leiomyosarcoma (28%), and sarcoma not otherwise specified (5%); “others” comprised 33% of the study population (Figure 1). Most tumors were high grade by either the FNCLCC or TNM two-tier grading system (57%). Only 4% of tumors were nodal-positive (*N*1 disease), and 3% were multifocal. The median tumor size was 13.9 cm (IQR: 8.2–21.0). Resections typically included en bloc resection of 1-2 organs (55%), most commonly left colon (27%), left kidney (23%), or small bowel (27%). A small proportion of patients were treated with radiation therapy (8%), chemotherapy (3%), or both radiation and chemotherapy (19%). The majority of resections were *R*0 (57%); however, *R*1 and *R*2 resections occurred in 32% and 8% of cases, respectively.

### 3.2. Disease-Free Survival

**Figure 2:**
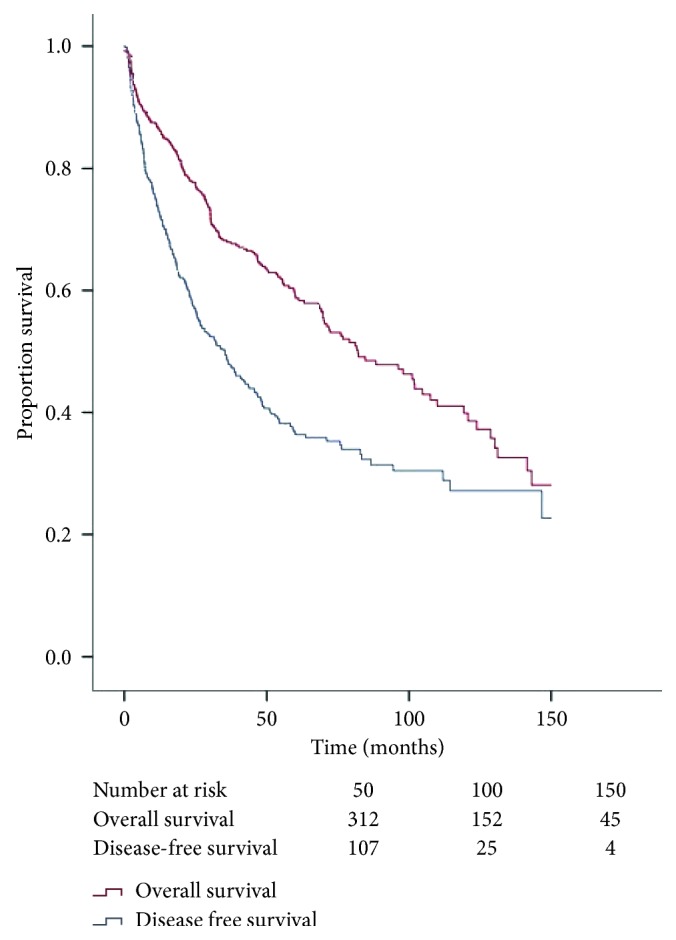
Kaplan–Meier curves for disease-free and overall survival demonstrating median survival of 35.3 months (95% CI: 27.6–43.0) and a 1-, 5-, and 10-year overall survival of 86.2%, 64.3%, and 58.7%, respectively, with associated number at risk for each time point.

**Figure 3:**
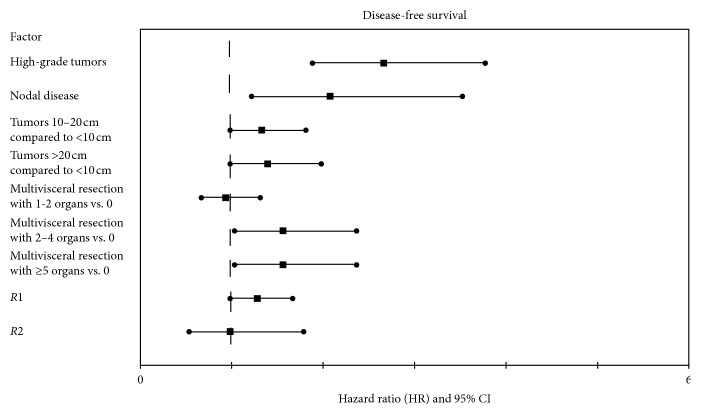
Forest plot demonstrating the hazard ratios and associated 95% confidence intervals for each factor included in the multivariate Cox proportional hazards model for disease-free survival.

Median disease-free survival was 35.3 months (95% CI: 27.6–43.0) (Figure 2). Univariate analysis showed tumor grade, nodal status, tumor size, number of organ resected, and margin status to be significantly associated (*p* < 0.05) with disease-free survival. Of these, high-grade tumors (*p* < 0.01; HR: 2.66, 95% CI: 1.88–3.77), nodal-positive disease (*N*1) (*p* < 0.01; HR: 2.08, 95% CI: 1.22–3.52), and larger en bloc resections (3-4 organs) (*p*=0.04; HR: 1.56, 95% CI: 1.03–2.37) were found to be independent predictors of disease-free survival on multivariate Cox proportional hazards analysis (Figure 3).

### 3.3. Overall Survival

**Figure 4:**
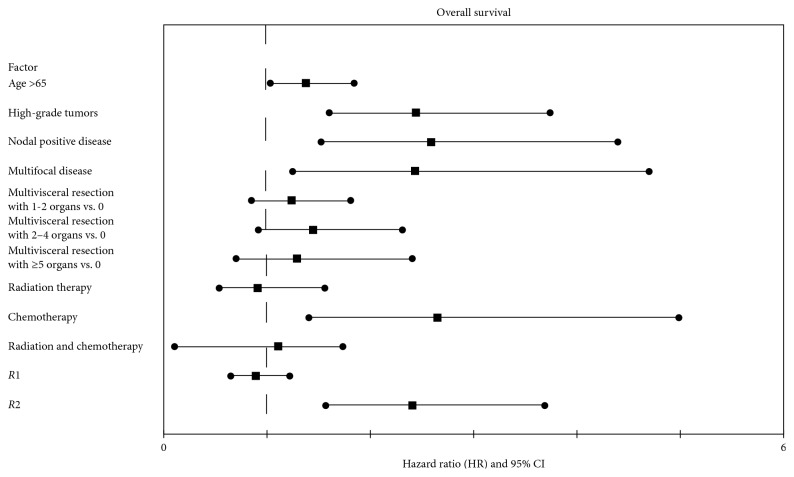
Forest plot demonstrating the hazard ratios and associated 95% confidence intervals for each factor included in the multivariate Cox proportional hazards model for overall survival.

Median overall survival was 81.6 months (95% CI: 66.3–96.8) (Figure 2). On univariate analysis, as shown in Figure 4, age greater than 65, tumor grade, nodal status, presence of multifocal disease, number of organs resected, receipt of additional therapies, and margin status were significantly associated (*p* < 0.05) with overall survival. Of these, multivariate Cox proportional hazards demonstrated that age greater than 65 (*p*=0.03; HR: 1.38, 95% CI: 1.03–1.84), high-grade tumors (*p* < 0.01; HR: 2.44, 95% CI: 1.60–3.74), nodal-positive disease (*N*1) (*p* < 0.01; HR: 2.59, 95% CI: 1.52–4.40), systemic chemotherapy (*p* < 0.01; HR: 2.65, 95% CI: 1.40–4.99), multifocal disease (*p* < 0.01; HR: 2.43, 95% CI: 1.25–4.69), and grossly positive margins (*R*2) (*p* < 0.01; HR: 2.41, 95% CI: 1.57–3.69) were found to be independently associated with worse survival.

### 3.4. Temporal Subanalysis

Disease-free survival and overall survival were compared across time to determine if differences in survival existed between different periods of treatment using 3-year bins (e.g., 2000–2003) examining 1-year outcome. No differences were found for either disease-free survival (*p*=0.37) or overall survival (*p*=0.19) for 1-year outcome on Chi-squared analysis.

### 3.5. Recurrence Pattern Subanalysis

Recurrence was analyzed by site, including distant, locoregional, or both, for the 237 patients for which data were available. For this cohort, median recurrence-free survival was 23.7 months. Median recurrence-free survival was found to be significantly different (*p*=0.02), with locoregional recurrence at 25.4 months (95% CI: 14.6–36.2), distant recurrence at 16.4 months (95% CI: 10.4–22.4), and both locoregional and distant recurrence at 36.4 months (95% CI: 23.7–49.1). Chi-squared analysis demonstrated no differences in recurrence patterns (locoregional versus distant recurrence versus both) and use of adjuvant radiotherapy (*p*=0.67), neoadjuvant radiotherapy (*p*=0.17), neoadjuvant chemotherapy (*p*=0.16), or adjuvant chemotherapy (*p*=0.64).

### 3.6. Histology Subanalysis

The most common histological subtypes included well-differentiated liposarcoma (*n* = 71), dedifferentiated liposarcoma (*n* = 69), and leiomyosarcoma (*n* = 162). Differences were detected on pooled logrank analysis for median recurrence-free survival between well-differentiated liposarcoma (49.1 months 95% CI: 26.0–72.1), dedifferentiated liposarcoma (26.9 months 95% CI: 12.7–41.4), and leiomyosarcoma (25.4 months 95% CI: 16.4–34.3) (*p*=0.03), as well as in median overall survival between well-differentiated liposarcoma (142.1 months 95% CI: 94.1–190.2), dedifferentiated liposarcoma (51.2 months 95% CI: 33.1–69.2), and leiomyosarcoma (70.8 months 95% CI: 56.8–84.8) (*p* < 0.01).

In a further subanalysis on patients with dedifferentiated liposarcoma, there was no difference detected on Chi-squared analysis between the recurrence pattern (locoregional versus distant recurrence versus both) and patients who received adjuvant radiotherapy (*p*=0.58), neoadjuvant radiotherapy (*p*=0.64), neoadjuvant chemotherapy (*p*=0.54), or adjuvant chemotherapy (*p*=0.13). There was no difference when comparing recurrence and patients who received adjuvant radiotherapy (*p*=1.00), neoadjuvant radiotherapy (*p*=1.00), neoadjuvant chemotherapy (*p*=0.71), or adjuvant chemotherapy (*p*=1.00). Finally, there was no difference between overall survival in patients who received adjuvant radiotherapy (*p*=0.51), neoadjuvant radiotherapy (*p*=0.14), neoadjuvant chemotherapy (*p*=0.10), or adjuvant chemotherapy (*p*=1.00).

In a similar subanalysis on patients with leiomyosarcomas, on Chi-squared analysis, there was no difference in the pattern of recurrence and patients who received neoadjuvant radiation therapy (*p*=1.00), neoadjuvant chemotherapy (*p*=0.71), or adjuvant chemotherapy (*p*=0.66). Additionally, there was no difference in recurrence in patients who received neoadjuvant radiation (*p*=0.64), adjuvant radiation (*p*=0.82), neoadjuvant chemotherapy (*p*=1.00), or adjuvant chemotherapy (*p*=0.47), as well as overall survival in those who received neoadjuvant radiotherapy (*p*=0.15), adjuvant radiotherapy (*p*=0.07), or adjuvant chemotherapy (*p*=1.00). In those that received adjuvant radiation therapy, there were less than predicted patients with locoregional recurrences as compared to distant metastases (0 versus 14 patients; *p*=0.04). Further, those patients which underwent neoadjuvant chemotherapy had an increased overall survival on Chi-squared analysis (44% versus 18% mortality; *p*=0.04). However, when accounting for factors previously shown to be important predictors of overall survival (Figure 4), including age, tumor grade, nodal status, presence of multifocal disease, degree of multivisceral resection, and margin status, neoadjuvant chemotherapy was not found to be an independent predictor of overall survival on multivariate logistical regression analysis (*p*=0.08; OR = 3.83; 95% CI: 0.84–17.5).

### 3.7. Chemotherapy and Radiation Therapy Analysis

A total of 12.3% (*n* = 70) patients underwent neoadjuvant radiation therapy, 15.6% (*n* = 89) underwent neoadjuvant chemotherapy, and 16.3% (*n* = 93) underwent adjuvant chemotherapy. On univariate analysis, recurrence was not significantly different between patients receiving neoadjuvant radiation therapy (*p*=0.69), neoadjuvant chemotherapy (*p*=0.35), or adjuvant chemotherapy (*p*=0.20).

Survival was not significantly different between patients receiving neoadjuvant radiation therapy (*p*=1.00), adjuvant radiation therapy (*p*=0.56), neoadjuvant chemotherapy (*p*=0.88), or adjuvant chemotherapy (*p*=0.65). Chemotherapy and radiation therapy practices did not change across time when examined in 3-year increments (e.g., 2000–2003) for neoadjuvant radiation therapy (*p*=0.23), adjuvant radiation therapy (*p*=0.59), neoadjuvant chemotherapy (*p*=0.11), or adjuvant chemotherapy (*p*=0.27). A total of 6.7% of patients (*n* = 38) underwent intraoperative radiation therapy (IORT). On Chi-squared analysis, there were no differences in rates of positive margins (*p*=0.40), recurrence patterns (locoregional versus distant versus both; *p*=0.67), or recurrence-free survival (*p*=1.00). Of those who received neoadjuvant radiation, there was no difference in the postoperative margin status (*p*=0.18).

## 4. Discussion

Retroperitoneal sarcomas (RPS) represent a small subset of all soft-tissue sarcomas [1]. In addition, there are numerous histiotypes, making these tumors a challenge to study, frequently without the sample size fit to draw substantial conclusions. This is problematic as RPS tend to present at late stages, with large tumor size and frequent recurrence (greater than 90% recurrence at 10 years has been reported) [17]. Therefore, an updated, multi-institutional study is useful to determine factors associated with survival and recurrence to better counsel patients on the risks and oncologic outcomes associated with resection of RPS. Our results demonstrate that tumor-specific factors are more associated with survival outcomes in RPS than patient- or provider-specific factors.

This contemporary study represents over 500 patients with primary RPS at 8 US tertiary referral centres with long-term follow-up, making this the largest multi-institutional North American study available for interpretation. A particular strength of this study, not possible with many large-scale administrative or registry databases, is the inclusion of recurrence data (in addition to our overall survival analysis). Disease-free survival was found to be a median of 35.3 months, while median overall survival was found to be 81.6 months, respectively. These data are comparable, or more favorable, to other large retrospective series or large database analyses [18, 19]. Distant recurrence was found to occur in a significantly shorter time interval (16.4 months) than either locoregional (25.4 months) or combined distant/locoregional (36.4 months).

High-grade tumors have been consistently shown to be a predictor of mortality and recurrence, with well-differentiated tumors of the same histiotype shown to have better long-term outcomes [5]. This has been reproducible and emphasizes its use in TNM staging, where grade has persisted despite recent updates in the AJCC staging system [20]. The number of patients in this cohort with high-grade tumors was 57%, matching the typical 2 : 1 high-grade to low-grade ratio described in prior studies [1]. The Transatlantic Working Group (TAWG) has taken this evidence so far as to recommend core-needle biopsies prior to resection to determine molecular subtype/grade when imaging is not pathognomonic [21].

Soft-tissue sarcomas are classically described as traveling hematogenously; however, a small proportion of tumors do metastasize to lymph nodes [1]. Our data show that 4% of patients have nodal metastases, found to be an independent predictor of both recurrence and mortality [5]. This is consistent with prior reports; these patients had a 2-fold incidence of recurrence and a 2.5-fold incidence of mortality. In total, 35% of those found with nodal disease were leiomyosarcoma, with the rest comprising numerous other histiotypes. However, despite the larger proportion of leiomyosarcoma seen with *N*1 disease, they still represent only 8 out of 157 cases. Therefore, this report and other reports of nodal significance are not likely to prompt prophylactic nodal dissections—or even sentinel lymph node biopsy—for sarcoma due to the rarity of its occurrence, but should be recognized on preoperative imaging and potentially sampled, if present, for risk stratification.

The goals of primary oncologic resection of retroperitoneal sarcomas are to achieve a complete resection of the tumor. However, these tumors present with an average size of approximately 12–23 cm [9, 22]. In this study, multifocal tumors contributed to worse overall survival. Additionally, multivisceral resections likewise decreased disease-free survival. Historically, the belief was that survival is dependent on obtaining at least an *R*1 resection, with some reports of improved overall survival with *R*0 as compared to *R*1 resection [6]. Our study corroborates prior findings that grossly positive margins (*R*2) were associated with decreased overall survival. Therefore, attempts to achieve a complete macroscopic resection should be striven for, when possible, recognizing that margins are one of the most modifiable factors associated with survival (HR-2.48). However, it should also be recognized that a *R*2 resection is oftentimes a surrogate for more aggressive tumor biology.

Klooster et al. found that those patients with an *R*2 resection tended to have decreased overall survival but that a subset did survive greater than 5 years [10], and that chemotherapy, but not radiation therapy, may have had a beneficial effect during the first three years following resection. Conversely, our study found no evidence for beneficial effects from either neoadjuvant or adjuvant chemotherapy on bi- and multivariate survival outcomes. On subgroup analysis of patients with leiomyosarcoma, neoadjuvant chemotherapy was associated with increased overall survival. However, it was not found to be significant on multivariate logistical regression analysis. In fact, for the entire cohort, chemotherapy was associated with worse overall survival (HR: 2.65) on Cox proportional hazards analysis. This likely represents the histiotype of tumors being treated with chemotherapy. The absence of benefit is consistent with other prior studies; however, recent high-quality prospective cohort studies and randomized control trials have shown some benefits to local recurrence-free and overall survival with both radiation therapy and neoadjuvant chemotherapy [12, 13, 19, 23, 24].

Radiation therapy has been also suggested to decreased locoregional recurrence. In a recent Transatlantic Retroperitoneal Working Group study of both well-differentiated and dedifferentiated liposarcoma, perioperative radiation therapy was associated with decreased locoregional recurrence on univariate but not multivariate analysis [25]. In a systematic review by Cheng et al., although radiation therapy was associated with a relatively favorable toxicity profile, some studies demonstrated a benefit on local recurrence and *R*0 resection rates which indirectly resulted in increased overall survival, while others did not [26]. In the univariate subanalysis of patients with leiomyosarcoma, rates of locoregional recurrence were decreased following adjuvant radiation therapy. However, on additional univariate analysis, postoperative margin status was not affected by neoadjuvant radiation therapy.

Differences in the studies described may be a reflection of retrospective studies mirroring practice as compared to controlled clinical trials. It will likely take time for retrospective studies to show the benefits described in clinical trials, if indeed those benefits are proven to be effective, rather than just efficacious. Interestingly, analysis of disease-free and overall survival across time shows that short-term (1 year) outcomes do not differ, suggesting therapies have not significantly changed short-term outcomes in clinical practice over the past 15 years. Additionally, we found that despite increasing interest in both neoadjuvant and radiation therapy in combined multimodal treatment, there have been no differences in utilization of chemotherapy or radiation therapy in this cohort. This trend could be explained by the relative rarity of these tumors and the poor historical outcomes associated with treatment of retroperitoneal sarcomas.

Despite its merits, there are limitations with our study. This retrospective cohort analysis over a long time period, with its inherent selection bias, has allowed for meaningful accrual of data but also represents a relatively heterogeneous group of tumor subtypes. Approximately one-third of patients had one of any number of less common retroperitoneal tumor histiotypes. This issue has been pervasive throughout the study of sarcomas and likely acts to limit the total sample size of these rare tumors, limiting statistical power. Unfortunately, it is unlikely that accrual of high-quality, level-one evidence addressing specific sarcoma histiotypes is possible, primarily due to the rarity of these tumors when considered as specific tumor types.

Finally, the rarity of sarcomas also requires pooling of data to include enough patients to draw meaningful conclusions, and institutional practice variability (e.g., timing of resection, use of locoregional, and systemic therapies) certainly exists despite having a similar patient pool. Some of this effect may be mitigated by including a large group of practices, and investigating differences between practice patterns as it relates to survival outcomes will be a project that could be studied in the future.

Sarcomas are a diverse and relatively rare tumor type in adults, often presenting as large masses with local invasion into surrounding structures, with complete surgical resection (*R*0/*R*1) the mainstay of treatment. Here, with a multi-institutional experience of resected RPS, we have shown that tumor-specific factors contribute more than patient-specific factors for both overall and disease-free survival. Additionally, nonsurgical therapies, such as chemotherapy and radiation therapy, seem to have no association with increased survival in our cohort. This appears to be a sustained trend across time. Further study using histiotype-specific databases should be developed to determine optimal treatment patterns for various retroperitoneal sarcomas.

## Figures and Tables

**Table 1:** Demographic and tumoral data for all patients included in the study.

Demographic/tumor characteristic	*N* (%)
Age >65	
Yes	196 (34)
No	375 (66)

Sex	
Male	255 (45)
Female	316 (55)

Race	
White	411 (72)
African American	69 (12)
Others/unknown	91 (16)

BMI	
≥30	141 (25)
<30	430 (75)

Smoking status	
Yes	496 (87)
No	75 (13)

Prior radiation exposure	
Yes	35 (6)
No	536 (94)

Known genetic syndrome	
Yes	556 (97)
No	15 (3)

Histology	
Leiomyosarcoma	162 (28)
Liposarcoma	196 (34)
Sarcoma NOS	26 (5)
Other sarcomas	187 (33)

Grade	
Low grade	131 (23)
High grade	326 (57)

Nodal disease	
*N*0	549 (96)
*N*1	22 (4)

Multifocal disease	
Yes	17 (3)
No	554 (97)

Size of tumor	
<10 cm	201 (35)
10–20 cm	217 (38)
>20 cm	145 (25)

Organs resected	
0	140 (25)
1-2	315 (55)
3-4	82 (14)
≥5	34 (6)

Therapies	
No therapy	396 (70)
Radiation therapy	45 (8)
Chemotherapy	19 (3)
Radiation and chemotherapy	111 (19)

Margin status	
*R*0	328 (57)
*R*1	184 (32)
*R*2	45 (8)

## Data Availability

The data used to support the findings of this study may be released upon application to the corresponding author via abbott@surgery.wisc.edu.
